# Social Determinants of Diabetes‐Related Foot Ulcer Healing and Amputation in Australia: A Systematic Review

**DOI:** 10.1002/jfa2.70107

**Published:** 2026-02-11

**Authors:** Tsz Long Chan, Sean Sadler, Angela Searle, Kym Hennessy, Sean Lanting, Vivienne Chuter

**Affiliations:** ^1^ Discipline of Podiatry, School of Health Sciences Western Sydney University Campbelltown New South Wales Australia

**Keywords:** access of health care, amputation, diabetes‐related foot ulcers, remoteness, social determinants of health, socio‐economic status, wound healing

## Abstract

**Introduction:**

In Australia, approximately 50,000 people suffer from diabetes‐related foot ulcers (DFU) with another 300,000 people at risk each year. Evidence suggests that social determinants of health may impact DFU outcomes; however, systematic evaluation of studies is lacking. The aim of this systematic review was to evaluate studies investigating the impact of social determinants of health on DFU outcomes.

**Methods:**

MEDLINE, EMBASE, CINAHL and Scopus were searched from inception to December 2024. Original published studies conducted in Australia investigating the impact of social determinants of health on DFU outcomes, such as wound healing, hospital admission and amputation, were potentially eligible for inclusion. Social determinants of health had to meet the World Health Organisation definition. Authors independently screened studies for inclusion, extracted data and assessed risk of bias using the Quality in Prognostic Studies tool.

**Results:**

Twelve studies which investigated socioeconomic status (*n* = 5), geographical remoteness (*n* = 5) and access to healthcare services (*n* = 7) and their impact on DFU outcomes were included. Socioeconomic disadvantage and geographic remoteness were associated with worse DFU outcomes including longer healing times and increased rates of amputation. Access to health services, which aligned with guideline recommendations, was associated with improved DFU healing outcomes.

**Conclusions:**

Social determinants of health impact DFU outcomes and are important for clinicians, policy makers and researchers to consider in the design and delivery of health care services. Further research is needed to explore the impact of social determinants of health on a broader range of DFU outcomes and across diverse geographical settings.

**Abbreviations**
CI: Confidence intervals
DFD: Diabetes‐related foot disease
DFU: Diabetes‐related foot ulcers
HRFS: High risk foot service
IRSD: Index of relative socioeconomic disadvantage
LGA: Local government area
MMM: Modified Monash Model
N/A: Not applicable
PAD: Peripheral artery disease
QUIPS: Quality in prognostic studies
SDHs: Social determinants of health
SEIFA: Socioeconomic indexes for areas
WHO: World Health Organization

## 1 Introduction

In 2021, there were 537 million people affected by diabetes and this figure is expected to surge to 783 million by 2045 [1]. In Australia, 1.5 million people, or approximately 5.2% of the total population, have diabetes [2]. A common complication of diabetes is diabetes‐related foot ulcers (DFU) [3]. DFU are typically associated with the presence of peripheral artery disease (PAD) and/or peripheral neuropathy and are estimated to affect up to 34% of people with diabetes in their lifetime [4]. Approximately half of all DFU become infected, with both infection and PAD increasing the likelihood of non‐healing or amputation [5]. Each day in Australia, up to 50,000 people live with a DFU, with six times that number at risk of developing a DFU [6, 7]. Annually in Australia, the amount spent on managing DFU and its complications is estimated to be over $1.6 billion [8].

Significant research has been conducted to identify factors that contribute to the development of DFU and the associated complications such as delayed healing, hospital admission and amputation. Studies have identified medical factors, such as PAD, DFU infection, increasing diabetes duration and poor glycaemic control, increase the risk of DFU and its complications [3, 9, 10, 11, 12]. There is a growing body of evidence examining the impact that social determinants of health (SDH) have on DFU outcomes, such as DFU healing time and rates of amputation [13, 14, 15]. The World Health Organization (WHO) defines SDH as ‘non‐medical factors that influence health outcomes’ [16]. Examples of SDH include socioeconomic status, education, employment status, access to healthcare and housing and living circumstances [16].

Globally it has been demonstrated that being older in age, from English speaking countries and from a lower household income family increase the risk of DFU development and DFU nonhealing [15, 17]. In the Australian context, the Australian Guidelines for diabetes‐related foot disease, released in 2021, identified that access to timely and culturally safe footcare for people living with diabetes remains a key challenge [18]. SDH, such as lack of availability of affordable health care services, socioeconomic depravation and food insecurity have been implicated in poorer health outcomes for people living in Australia, including those living with diabetes and DFU [14, 18, 19]. However, systematic evaluation of these studies is lacking. Therefore, the aim of this systematic review was to evaluate studies investigating the impact of SDH on DFU outcomes.

## 2 Materials and Methods

This systematic review has been reported in accordance with the Preferred Reporting Items for Systematic Reviews and Meta‐Analyses (PRISMA) checklist [20] and was prospectively registered with PROSPERO (CRD42023403310).

### 2.1 Eligibility Criteria

Only studies conducted in Australia that included adults (18 years of age and older) with a current DFU were eligible for inclusion. Any quantitative original study reporting the impact of SDH on DFU outcomes, including but not limited to healing, hospital admission, major amputation, limb salvage (including minor amputation and re‐amputation) and amputation‐free survival, was eligible for inclusion. SDH had to meet the WHO definition with examples of SDH including income and social protection; education; unemployment and job security; working life conditions; food insecurity; housing, basic amenities and the environment; early childhood development; social inclusion and nondiscrimination; structural conflict and access to affordable health services of decent quality [16]. Studies that reported SDH or DFU outcomes as part of mixed populations, for example, combined with people with diabetes but not DFU, were ineligible. Studies solely investigating the effect of medical conditions (including PAD, infection and psychosocial conditions) or medical interventions (such as dietary supplementation) on DFU outcomes were also ineligible.

### 2.2 Search Strategy

MEDLINE, EMBASE, CINAHL and Scopus electronic databases were searched from their inception to 1^st^ December 2024. Truncated search terms were used to ensure that relevant studies were included (Table 1). Titles and abstracts were exported to EndNote20 for screening. Additional hand searches of reference lists of included articles and published review articles were conducted.

**TABLE 1:** Search terms.

Databases: MEDLINE, EMBASE, CINAHL and Scopus	
1	(foot adj5 ulcer*).ti,ab.
2	(feet adj5 ulcer*).ti,ab.
3	foot disease
4	(chronic limb‐threatening isch?emia or critical limb isch?emia or limb isch?emia).mp
5	1 or 2 or 3 or 4
6	heal*.mp.
7	limb salvage.ti,ab.
8	amputation.mp. or amputation/
9	6 or 7 or 8
10	5 and 9

### 2.3 Study Selection and Data Extraction

All titles and abstracts retrieved were independently assessed by two reviewers (TLC and AS). Disagreements were resolved by consensus between the two authors and where required a third reviewer (VC or SS). Reviewers (TLC and AS or VC) independently extracted data related to study characteristics, participant demographics, SDH and DFU outcomes reported in included studies using a predesigned data extraction form. Data planned to be extracted included participant characteristics, SDH and outcomes related to DFU such as hospital data, healing, amputation, limb salvage and survival and mortality outcomes. Meta‐analysis was planned to be conducted where possible for DFU outcomes such as healing versus nonhealing and amputation versus no amputation by SDH (e.g., socioeconomic status or remoteness).

### 2.4 Quality Assessment

Included full texts were independently appraised by three reviewers (TLC, KH and SS) for risk of bias using the Quality In Prognostic Studies (QUIPS) tool [21]. This tool has six domains including study participation, study attrition, prognostic factor measurement, outcome measurement, study confounding and statistical analysis and reporting. Each domain is rated yes, partial, no, or unsure and an overall judgement of the risk of bias (high, moderate or low) within each domain is made based on the ratings of the included items. There were no restrictions on the level of quality required for inclusion in this systematic review. No disagreements occurred so arbitration by a fourth independent reviewer (VC or SL) was not required. No reviewer extracted data from or appraised a study of which they are an author.

## 3 Results

**FIGURE 1:**
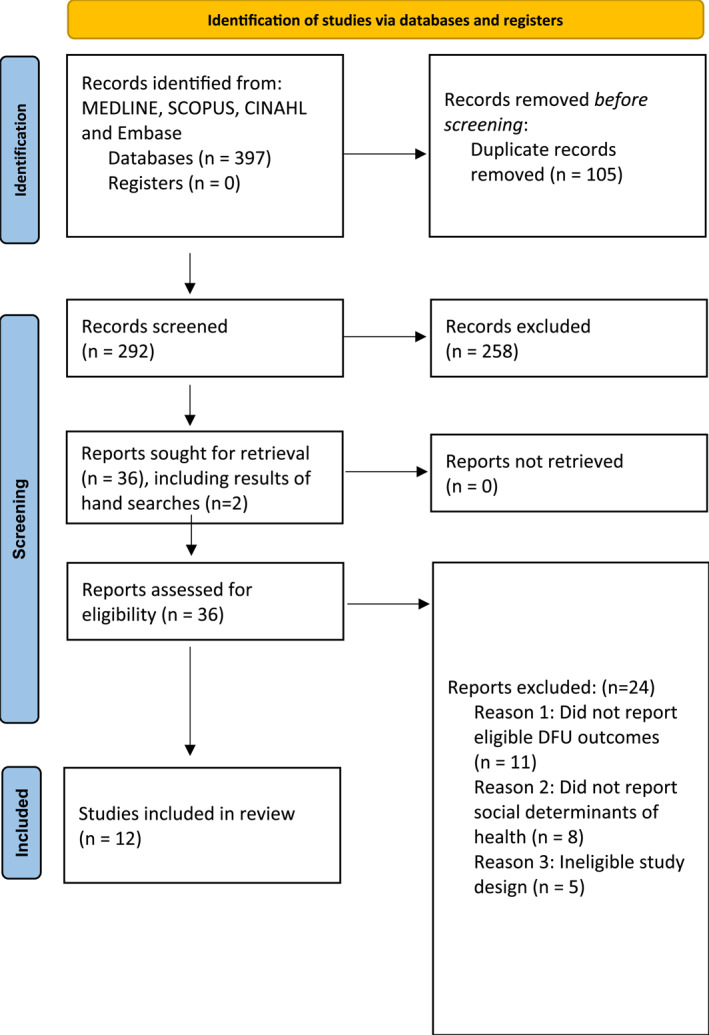
PRISMA flow chart.

A total of 292 titles and abstracts were retrieved (after removal of duplicates), of which 36 were suitable for full‐text review. Twenty‐four papers were excluded for the following reasons: did not report eligible DFU outcomes (*n* = 11), did not report SDH (*n* = 8) or ineligible study design (*n* = 5) (Figure 1).

### 3.1 Characteristics and Overview of Included Studies

Twelve studies were deemed eligible for inclusion in this systematic review. Nine of the studies used a retrospective design [22, 23, 24, 25, 26, 27, 28, 29, 30], two studies were secondary analyses of prospective studies [31, 32] and one was a prospective study [33] (Table 2). All studies reported the diabetes population percentage, ranging from 2.8% to 100% and six studies reported First Nations status, ranging from 3.4% to 51.7% [23, 25, 26, 27, 31, 32] (Table 2). SDH reported included socioeconomic status (*n* = 5) [22, 28, 29, 31, 33], geographical remoteness (*n* = 5) [23, 27, 30, 31, 32] and aspects related to access to health care services (*n* = 7) [24, 25, 26, 27, 28, 29, 32] (Table 2).

**TABLE 2:** Study characteristics of included studies.

Study	Study type, number of centres, study period and patient selection	No. people/limbs, diabetes % and First Nations %	Social determinants	How social determinants are measured	Outcome measures	Follow‐up
Alahakoon et al. (2024) [30]	Retrospective, Townsville University Hospital between 1 Jan 2000 and 31 Dec 2020, convenience sampling	*N* = 534 (toe amputations 94.4%; trans‐metatarsal amputation 4.5% and mid‐tarsal amputation 1.1%) DM: 100% First Nations: 27.0%	Remoteness	MMM	Major amputation (proximal to ankle)	Medial follow‐up 4.0 years (IQR 2.1–7.6)
Bergin et al. (2011) [22]	Retrospective, Victorian Admitted Episodes Database for years 2004/05 and 2005/06, convenience sampling	Group D (IRSD 1/2): 2268 DM: 3.8% Group A (IRSD 9/10): 2734 DM: 2.8% First Nations: NS	Socioeconomic status	IRSD	Amputation	NS
Jeyaraman et al. (2019) [23]	Retrospective, Royal Darwin Hospital HRFC between Jan 2003 and Jun 2015, convenience sampling	*n* = 513 DM: 100% First Nations: 48.1%	Remoteness	Did not specify how to classify remote areas	Amputation	Median follow up: 5.8 years (IQR: 3.1–9.8)
Jia et al. (2017) [31]	Retrospective, secondary analysis of another study in QLD 13/17 hospital diabetic foot service between Jan 2012 and Dec 2013, convenience sampling	*n* = 853 DM: 100% First Nations: 13.6%	Socioeconomic status	IRSD	Ulcer healing	12 months follow up
Linton et al. (2021) [24]	Retrospective audit, central coast local health district patients with diabetes underwent minor foot amputation within 2017, convenience sampling	*n* = 74, DM: 100% First Nations: NS	Access of health care service	Medical Record	Minor amputation	12 months from their amputation
Macfarlane et al. (2024) [25]	Retrospective cohort study in Gosford Hospital, 2017 and 2019, convenience sampling	*n* = 120 with 141 ulcers DM: 100% First Nations status: 2017: 4.9% 2019: 3.4%	Access of healthcare service	Medical record	Ulcer healing and amputation	NS
Manewell et al. (2022) [26]	Retrospective, 2 public Principal referral hospital within Sydney local health district between Jul 2012 and Jun 2018, convenience sampling	*n* = 564 DM: 100% First Nations: 5%	Access of healthcare service	Hospital data	Minor and major amputation, revascularisation and DFU related hospital admission	NS
O'Rourke et al. (2013) [27]	Retrospective, Cairns Base Hospital between 1998 and 2008, convenience sampling	*N* = 143 DM: 100% First Nations: 51.7%	Remoteness and access of healthcare service	Place of usual residence on medical records	Amputation	NS
Soonarane et al. (2024) [28]	Retrospective, hospitals in metro south health, SE QLD between Jan 2018 and Dec 2021, convenience sampling	Amputated limbs *N* = 446 DM: 100% First Nations: NS	Access of healthcare services and socioeconomic status	Medical record SEIFA	Amputation, death and hospital discharge	NS
Tehan et al. (2023) [33]	Prospective, Liverpool, Gosford, Wyong and John Hunter Hospital between 2017 and 2020, convenience sampling	*N* = 117 DM: 100% First Nations: NS	Socioeconomic status	IRSD	Ulcer healing	6 months
Tehan et al. (2022) [29]	Retrospective, Hunter New England LHD–Data from medical records 2014–2018, convenience sampling	*N* = 476 DM: 66% First Nations: NS	Socioeconomic status and remoteness	IRSD and distance from Newcastle city	Ulcer healing outcome and amputation	12 months
Zhang et al. (2022) [32]	Retrospective, secondary analysis of 15 of 17 hospital and health service regions cohort study in QLD between July 2011–Dec 2017, convenience sampling	*N* = 4709 DM: 100% First Nations: 10.5%	Remoteness, access of healthcare service	Place of residence	Ulcer healing outcome and amputation	24 months

Abbreviations: DFU, diabetes‐related foot ulcer; DM, diabetes mellitus; ESRF, end stage renal failure; IQR, interquartile range; IRSD, index of relative socioeconomic disadvantage; LEA, lower extremity amputation; MMM, modified Monash model; NS, not stated and SEIFA, socioeconomic indexes for areas.

The majority of studies included a predominantly older male population (i.e., > 60 years of age) [22, 25, 26, 27, 29, 30, 31, 32, 33] (Table 3). Prevalence of chronic health conditions and participant characteristics are reported in Table 3. Half of included studies used a wound classification system including the University of Texas classification systems (*n* = 4) [25, 31, 32, 33], Wagner grading scale (*n* = 1) [23] and WIfI risk of amputation (*n* = 1) [29] (Table 3). DFU outcomes per SDH are reported in Table 4.

**TABLE 3:** Patient demographics of included studies.

Study	Mean age years (SD)	Male %	PAD %	Kidney diseases %	Coronary disease %	Hypertension %	Smoking history %	Lesion and wound classification
Alahakoon et al. (2024) [30]	61.61 (53.0–71.0)	*N* = 370 (69.3%)	*N* = 190 (33.3%)	End stage renal failure: *n* = 54 (10.1%)	Ischaemic heart disease: *n* = 217 (40.6%)	*N* = 391 (73.2%)	Current smoker: *n* = 282 (52.8%)	NS
Bergin et al. (2011) [22]	Group D (IRSD 1 or 2) Male: 53.0 and female: 69.0 Group A (IRSD 9 or 10): Male: 68.7 and female: 73.6	Group D: 66.2%; Group A: 81.0%	Group D: 42.9%; Group A: 45.3%	NS	NS	NS	NS	NS
Jeyaraman et al. (2019) [23]	Total: 56.6 (12.5) Age at LEA: First Nations amputees: 52.9 (10.3) Non‐Indigenous amputees: 61.9 (11.8)	Total: 62.8% First Nations amputees: 52.4% Non‐Indigenous amputees: 83.9%	Total: 42.6% First Nations amputees: 41.4% Non‐Indigenous amputees: 60.7%	Total: 48.0% First Nations amputees: 67.2% Non‐Indigenous amputees: 38.3%	Total: 33.9% First Nations amputees: 34.8% Non‐Indigenous amputees: 33.1%	Total: 89.0% First Nations amputees: 88.9% Non‐Indigenous amputees: 93.2%	Total: 55.0% First Nations amputees: 70.0% Non‐Indigenous amputees: 45.8%	Wagner grade ≥ 2: 54.8% Wagner grade 4: 1% Wagner grade 5: 0%
Jia et al. (2017) [31]	62.9 (12.8)	*N* = 575 (68.0%)	*N* = 330 (45.8%)	*N* = 60 (7.0%)	*N* = 90 (10.6%)	*N* = 211 (24.7%)	*N* = 37 (4.3%)	Ulcer area (mm^2^ (SD)): 235.7 (610.4) Texas wound classification 2/3: 66 (7.8%)
Linton et al. (2021) [24]	> 60 years of age: 78%	77%	NS	32.4%	58.1%	NS	23.0%	NS
Macfarlane et al. (2024) [25]	2017: 66.0 (11.8) 2019: 64.2 (12.3)	2017: *n* = 47 (77%) 2019: *n* = 50 (85%)	NS	Mean eGFR (SD) 2017: 59.3 (26.6) 2019: 63.5 (25.7)	NS	2017: *n* = 43 (70%) 2019: *n* = 43 (73%)	Current smoker 2017: *n* = 10 (16%) 2019: *n* = 12 (20%) Ex‐smoker 2017: *n* = 20 (33%) 2019: *n* = 17 (29%)	Texas wound classification 2017: 1: *n* = 82 (75%) 2: *n* = 8 (7.3%) 3: *n* = 9 (8.3%) 2019: 1: *n* = 41 (42%) 2: *n* = 12 (12%) 3: *n* = 17 (17%) *p* = 0.002 2017: A: *n* = 76 (70%) B: *n* = 15 (14%) C: *n* = 5 (4.6%) D: *n* = 3 (2.8%) 2019: A: *n* = 34 (35%) B: *n* = 16 (16%) C: *n* = 18 (18%) D: *n* = 1 (1%) *p* < 0.001
Manewell et al. (2022) [26]	69.5 (13.7)	71%	NS	Dialysis: 32 (6%)	NS	NS	Current smoker 60 (11%)	NS
O'Rourke et al. (2013) [27]	Total: 63.3 (14.0) First Nations: 56.3 (11.7) Non‐Indigenous: 70.9 (12.3) *p* = 0.000	Total: 58% First Nations: 49% Non‐Indigenous: 68% *p* = 0.018	Total: 25% First Nations: 38% Non‐Indigenous: 1.1% *p* = 0.000	Chronic kidney disease (stage 3,4 and 5) Total: 39% First Nations: 64% Non‐Indigenous: 13% *p* = 0.000	NS	NS	NS	NS
Soonarane et al. (2024) [28]	NS	NS	NS	NS	NS	NS	NS	NS
Tehan et al. (2023) [33]	63.35 (12)	82%	NS	NS	Myocardial infarction: 24% Stroke: 9% Deep vein thrombosis: 5%	NS	Current: 8% Previous: 45%	University of Texas 1A: 54%
Tehan et al. (2022) [29]	67 (13)	73%	NS	Renal failure: 12%	Cardiovascular disease: 78%	NS	Current: 14% Previous: 45%	WIfI risk of amputation (medium/high): 15.8%
Zhang et al. (2022) [32]	63 (IQR 54–72)	69.5%	None: 58.3% Mild to moderate: 35.7% Critical: 6.0%	Chronic kidney disease: 13.2% End stage kidney failure: 3.9%	20.9%	53.1%	Smoker: 10.5%	University of Texas 2/3: 15.6%

Abbreviations: DFU, diabetes‐related foot ulcer; IQR, interquartile range; IRSD, index of relative socioeconomic disadvantage; LEA, lower extremity amputation; NS, not stated; PAD, peripheral artery disease; SD, standard deviations; WIfI, wound, ischaemia, foot infection.

**TABLE 4:** Impact of social determinants of health on outcomes of DFU healing, amputation, limb salvage and mortality.

Study	Hospital data	DFU healing outcomes	Amputation outcomes	Limb salvage (LS)	Survival and mortality outcomes
Socioeconomic disadvantages					
Bergin et al. (2011) [22]	Total separations Group A: 2734 (62.3/1000 people with diabetes) Group D: 2268 (75.3/1000 people with diabetes) Individuals in more socio‐advantaged regions tends to have more separation from hospital. Which can mean they can afford going to hospital or they are more likely to have DFU needing hospitalised.	Cellulitis (per capita separations) Group A: 3.4 Group D: 4.3 Rate ratio (95% CI): 1.24 (0.1, 1.6) Rate of having cellulitis following DFU is lower in people living in less socioadvantaged regions Osteomyelitis (per capita separations) Group A: 5.2 Group D: 5.4 Rate ratio (95% CI): 1.04 (0.8, 1.3) Rate of having osteomyelitis following DFU is lower in people living in less socio‐advantaged regions.	Foot amputation (per capita separations) Group A: 6.9 Group D: 5.4 Rate ratio (95% CI): 0.8 (0.7, 1.0) Below knee amputation (per capita separations) Group A: 4.1 Group D: 7.4 Rate ratio (95% CI): 1.8 (1.5, 2.2) Above knee amputation (per capita separations) Group A: 1.8 Group D: 2.4 Rate ratio (95% CI): 1.35 (0.1, 1.9) Rate of having foot, below or above knee amputation is higher in people living in less socio‐advantaged regions.	NS	NS
Jia et al. (2017) [31]	NS	Factors for developing a foot infection: Socioeconomic status: Most disadvantaged: 67 (23.5%) Least disadvantaged: 26 (9.1%) *p* = 0.276	NS	NS	NS
Soonarane et al. (2024) [28]	NS	NS	SEIFA in 2021 Logan 944 Brisbane 1045 Redland 1024 After population size adjusted, mean number of amputations (per 100,000 people) Logan: 14 Brisbane: 8 Redland: 7	NS	NS
Tehan et al. (2023) [33]	NS	Ulcer healing: Healed IRSD: 978.14 (37) (less disadvantaged) Non‐healed IRSD: 955.76 (64) (more disadvantaged) *p* = 0.01 OR (95% CI) 1.01 (1.01–1.02) and *p* = 0.04	NS	NS	NS
Tehan et al. (2022) [29]	NS	NS	Amputation: No amputation IRSD: 974 (51) (relatively disadvantaged) Amputation IRSD: 970 (50) (relatively disadvantaged) *p* = 0.60	NS	NS
Remoteness					
Alahakoon et al. (2024) [30]	NS	NS	Major amputation: *n* = 103 (19.3%)–[ilsilateral side *n* = 84 (81.65%); contralateral side *n* = 19 (18.4%)] Repeated minor amputation: *n* = 230 (43.1%) There is no significant differences in the rate of major amputation, minor amputation or mortality between participants from regional cities.	NS	Died during follow up: *n* = 250 (46.8%)
Jeyaraman et al. (2019) [23]	NS	NS	Remote area of residence: Total LEA: 93/263 (35.5%) First Nations LEA: 82/145 (56.5%) Non‐Indigenous LEA: 11/118 (9.3%) *p* < 0.001	NS	NS
Jia et al. (2017) [31]	NS	Foot infection: Total: 288/720 (40.0%) Geographic remoteness: Major city: 131 (46.0%) Very remote area: 15 (5.3%) *p* = 0.434	NS	NS	NS
O'Rourke et al. (2013) [27]	NS	NS	Rural/remote location of amputation patient: First Nations: 48 (65%) Non‐Indigenous: 34 (49%) *p* = 0.060 Remote location of amputation patient: First Nations: 37 (50%) Non‐Indigenous: 5 (7.2%) *p* = 0.000	NS	NS
Zhang et al. (2022) [32]	NS	Median time‐to‐ulcer‐free (days (95% CI)) Total: 112 (105–119) Major city: 88 (84–98) Regional area: 140 (126–154) Remote: 315 (184–659) Longer time to ulcer free: major city versus. regional area hazard ratio (95% CI) 0.73 (0.68–0.79) Major city versus. remote area hazard ratio (95% CI) 0.51 (0.41–0.64) Adjusted probability of being ulcer‐free by 6 months (% (95% CI)): Major city: 65.0% (63.3–66.7) Regional area: 54.6% (52.6–56.8) Remote area: 40.3% (34.6–47.1)	NS	NS	NS
Access of healthcare service					
Linton et al. (2021) [24]	Limited access HRFC (1 site): Preamputation attendance: 12 (16%) Postamputation attendance: 13 (18%) Greater access wound clinics (2 sites): Preamputation attendance: 28 (38%) Post‐amputation attendance: 44 (60%)	NS	NS	NS	NS
Macfarlane et al. (2024) [25]	Patients admitted to hospital due to complications 2017: *n* = 28 (46%) 2019: *n* = 32 (54%)	% Of wound healed at 52 weeks 2017: *n* = 40 (58%) 2019: *n* = 49 (68%) Time to ulcer healed (weeks) 2017: 20.4 2019: 14.2 *p* = 0.021 % Difference in wound area (mm^2^) in incompletely healed ulcers 2017: 28.7% 2019: 19.1% Incidence of surgical debridement 2017: *n* = 13 (21%) 2019: *n* = 9 (15%) Requiring antibiotics 2017: *n* = 38 (62%) 2019: *n* = 44 (75%)	Incidence of amputation 2017: *n* = 11 (18%) 2019: *n* = 19 (32%) Proportion of amputation severity Minor 2017: *n* = 10 (91%) 2019: *n* = 17 (89%) Major/both 2017: *n* = 1 (9.1%) 2019: *n* = 2 (11%)	Incidence of vascular intervention 2017: *n* = 1 (1.6%) 2019: *n* = 11 (19%) *p* = 0.002	Deceased 2017: *n* = 20 (33%) 2019: *n* = 10 (17%) *p* = 0.045
Manewell et al. (2022) [26]	DFU related hospital admission: Known to HRFS: 103 (18%) Known to podiatry: 52 (9%)	NS	Minor amputation: Known to HRFS: 29 (19%) OR (95% CI): 1.04 (0.65–1.68), *p* = 0.86 Known to podiatry: 13 (8%) OR (95% CI): 0.87 (0.45–1.67), *p* = 0.67 Major amputation: Known to HRFS: 1 (3%) OR (95% CI): 0.14 (0.02–1.05), *p* = 0.06 Known to podiatry: 1 (3%) OR (95% CI): 0.32 (0.04–2.36), *p* = 0.26	NS	NS
O'Rourke et al. (2013) [27]	NS	NS	Attended HRFS prior to amputation: Total: 25 (17%) First Nations: 9 (12%) Non‐Indigenous: 16 (23%) *p* = 0.208	NS	NS
Soonarane et al. (2024) [28]	NS	NS	Amputation performed outside the residing LGAs Logan: *n* = 144 (73%) Brisbane: *n* = 7 (3%) Redland: *n* = 40 (95%) Redland has a smaller population where Logan has a large population but majority of their amputations were done outside the LGA	NS	NS
Tehan et al. (2022) [29]	NS	NS	Mean geographical distance from HRFS: Amputation: 107 (366) No amputation: 30 (40) *p* = 0.14 Pearson coefficient: *r* = 0.19 and *p* < 0.001 Odds ratio (95% CI) for 50 km distance increase from HRFS: 1.14 (1.02–1.24), *β* = 1.15 and *p* = 0.02	NS	NS
Zhang et al. (2022) [32]	NS	Median time‐to‐ulcer‐free (days (95% CI)): Recent DFU treatment by podiatrist: 111 (104–117) Nil treatment by podiatrist: 193 (145–257) *p* < 0.05 Shorter time to ulcer free: Recent DFU treatment by podiatrist: hazard ratio (95% CI) 1.31 (1.10–1.57)	NS	NS	NS

Abbreviations: CI, confidence interval; DFU, diabetes‐related foot ulcer; HRFS, high risk foot service; LEA, lower extremity amputation; NS, not stated; SEIFA, socioeconomic indexes for areas.

Due to heterogeneity of study outcomes and SDH reporting, and low numbers of included studies, meta‐analysis by SDH type was not possible.

### 3.2 Critical Appraisal of the Included Articles

The quality of the included studies varied with regards to the study participation, prognostic factor measurement, outcome measurement, study confounding and statistical analysis and reporting with no study found to have a low risk of bias in all six domains (Table 5). Study attrition was assessed in eight studies [23, 24, 26, 28, 29, 31, 32, 33]. This domain was considered not applicable for the remaining four studies because two did not report any exclusion of participants [22, 27] and the other two excluded participants with missing data at the beginning of the study [25, 30]. The majority of studies were rated as having a high risk of bias (*n* = 5) [22, 25, 26, 27, 28] or moderate risk of bias (*n* = 4) [23, 24, 29, 33] due to data related to potential confounders not being reported or considered in statistical analyses.

**TABLE 5:** Quality in Prognostic Studies (QUIPS) tool assessment table.

		[30]	[22]	[23]	[31]	[24]	[25]	[26]	[27]	[28]	[33]	[29]	[32]
1. Study participation	Goal: To judge the risk of selection bias (likelihood that relationship between *PF* and *outcome* is different for participants and eligible non‐participants).												
Source of target population	The source population or population of interest is adequately described for key characteristics (LIST).	Y	P	Y	Y	Y	Y	P	Y	Y	Y	Y	Y
Method used to identify population	The sampling frame and recruitment are adequately described, including methods to identify the sample sufficient to limit potential bias (number and type used, e.g., referral patterns in health care).	Y	P	Y	Y	Y	Y	Y	Y	Y	Y	Y	Y
Recruitment period	Period of recruitment is adequately described.	Y	P	Y	Y	Y	Y	Y	P	Y	P	P	Y
Place of recruitment	Place of recruitment (setting and geographic location) are adequately described.	Y	U	Y	P	Y	Y	P	Y	Y	Y	Y	Y
Inclusion and exclusion criteria	Inclusion and exclusion criteria are adequately described (e.g., including explicit diagnostic criteria or ‘zero time’ description).	Y	P	Y	Y	Y	Y	Y	Y	N	Y	Y	Y
Adequate study participation	There is adequate participation in the study by eligible individuals.	Y	U	Y	Y	Y	Y	Y	U	U	N	Y	U
Baseline characteristics	The baseline study sample (i.e., individuals entering the study) is adequately described for key characteristics (LIST).	Y	P	Y	Y	Y	Y	P	Y	N	Y	Y	Y
Summary study participation	The study sample represents the population of interest on key characteristics, sufficient to limit potential bias of the observed relationship between PF and outcome.	*L*	*H*	*L*	*M*	*L*	*L*	*M*	*M*	*H*	*M*	*L*	*L*
2. Study attrition	Goal: to judge the risk of attrition bias (likelihood that relationship between *PF* and *outcome* are different for completing and noncompleting participants).												
Proportion of baseline sample available for analysis	Response rate (i.e., proportion of study sample completing the study and providing outcome data) is adequate.	N/A	N/A	Y	Y	Y	N/A	Y	N/A	Y	Y	N	Y
Attempts to collect information on participants who dropped out	Attempts to collect information on participants who dropped out of the study are described.	N/A	N/A	Y	Y	Y	N/A	N	N/A	Y	U	U	U
Reasons and potential impact of subjects lost to follow‐up	Reasons for loss to follow‐up are provided.	N/A	N/A	Y	Y	Y	N/A	N	N/A	Y	N	U	Y
Outcome and prognostic factor information on those lost of follow‐up	Participants lost to follow‐up are adequately described for key characteristics (LIST).	N/A	N/A	Y	Y	Y	N/A	U	N/A	Y	N	N	N
	There are no important differences between key characteristics (LIST) and outcomes in participants who completed the study and those who did not.	N/A	N/A	U	Y	Y	N/A	U	N/A	U	U	U	U
Study attrition summary	Loss to follow‐up (from baseline sample to study population analysed) is not associated with key characteristics (i.e., the study data adequately represent the sample) sufficient to limit potential bias to the observed relationship between PF and outcome.	*N/A*	*N/A*	*M*	*L*	*L*	*N/A*	*H*	*N/A*	*M*	*M*	*H*	*M*
3. Prognostic factor measurement	Goal: to judge the risk of measurement bias related to how PF was measured (differential measurement of PF related to the level of outcome).												
Definition of the PF	A clear definition or description of 'PF' is provided (e.g., including dose, level, duration of exposure and clear specification of the method of measurement).	Y	Y	Y	Y	Y	P	Y	P	Y	Y	Y	Y
Valid and Reliable measurement of PF	Method of PF measurement is adequately valid and reliable to limit misclassification bias (e.g., may include relevant outside sources of information on measurement properties, also characteristics, such as blind measurement and limited reliance on recall).	Y	Y	Y	Y	Y	Y	Y	P	U	Y	Y	Y
	Continuous variables are reported or appropriate cut‐points (i.e., not data‐dependent) are used.	Y	Y	Y	Y	Y	Y	Y	Y	U	Y	Y	Y
Method and setting of PF measurement	The method and setting of measurement of PF is the same for all study participants.	Y	Y	Y	Y	Y	Y	Y	P	U	P	P	P
Proportion of data on PF available for analysis	Adequate proportion of the study sample has complete data for PF variable.	Y	Y	Y	Y	Y	Y	Y	Y	Y	Y	Y	Y
Method used for missing data	Appropriate methods of imputation are used for missing 'PF' data.	Y	U	Y	Y	Y	N	Y	U	U	U	U	Y
PF Measurement summary	*PF* is adequately measured in study participants to sufficiently limit potential bias.	*L*	*L*	*L*	*L*	*L*	*M*	*L*	*M*	*H*	*L*	*L*	*M*
4. Outcome measurement	Goal: to judge the risk of bias related to the measurement of outcome (differential measurement of outcome related to the baseline level of PF).												
Definition of the outcome	A clear definition of outcome is provided, including duration of follow‐up and level and extent of the outcome construct.	Y	Y	Y	Y	Y	Y	Y	Y	P	Y	Y	Y
Valid and Reliable measurement of outcome	The method of outcome measurement used is adequately valid and reliable to limit misclassification bias (e.g., may include relevant outside sources of information on measurement properties, also characteristics, such as blind measurement and confirmation of outcome with valid and reliable test).	Y	P	Y	Y	Y	P	Y	Y	P	Y	Y	P
Method and setting of outcome measurement	The method and setting of outcome measurement is the same for all study participants.	Y	P	Y	Y	Y	P	Y	P	U	Y	Y	P
Outcome measurement summary	*Outcome of interest* is adequately measured in study participants to sufficiently limit potential bias.	*L*	*M*	*L*	*L*	*L*	*M*	*L*	*L*	*H*	*L*	*L*	*L*
5. Study confounding	Goal: to judge the risk of bias due to confounding (i.e. the effect of PF is distorted by another factor that is related to PF and outcome).												
Important confounders measured	All important confounders, including treatments (key variables in conceptual model: LIST), are measured.	P	P	Y	Y	P	N	N	U	N	P	U	Y
Definition of the confounding factor	Clear definitions of the important confounders measured are provided (e.g., including dose, level and duration of exposures).	P	P	Y	Y	P	N	N	U	N	N	N	Y
Valid and Reliable measurement of confounders	Measurement of all important confounders is adequately valid and reliable (e.g., may include relevant outside sources of information on measurement properties, also characteristics, such as blind measurement and limited reliance on recall).	P	P	Y	Y	U	P	P	U	N	U	U	Y
Method and setting of confounding measurement	The method and setting of confounding measurement are the same for all study participants.	P	U	Y	Y	Y	Y	Y	U	U	U	U	P
Method used for missing data	Appropriate methods are used if imputation is used for missing confounder data.	Y	U	U	Y	Y	U	U	U	N	U	U	Y
Appropriate Accounting for confounding	Important potential confounders are accounted for in the study design (e.g., matching for key variables, stratification or initial assembly of comparable groups).	P	U	Y	Y	U	P	P	P	U	P	P	Y
	Important potential confounders are accounted for in the analysis (i.e., appropriate adjustment).	P	P	U	Y	U	N	P	N	N	P	P	Y
Study confounding summary	Important potential confounders are appropriately accounted for, limiting potential bias with respect to the relationship between *PF* and *outcome*.	*M*	*H*	*M*	*L*	*M*	*H*	*H*	*H*	*H*	*M*	*M*	*L*
6. Statistical analysis and reporting	Goal: to judge the risk of bias related to the statistical analysis and presentation of results.												
Presentation of analytical strategy	There is sufficient presentation of data to assess the adequacy of the analysis.	Y	Y	Y	Y	Y	Y	Y	Y	Y	Y	Y	Y
Model development strategy	The strategy for model building (i.e., inclusion of variables in the statistical model) is appropriate and is based on a conceptual framework or model.	Y	Y	Y	Y	P	P	Y	Y	U	Y	Y	Y
	The selected statistical model is adequate for the design of the study.	Y	Y	Y	Y	Y	Y	Y	Y	N	Y	Y	Y
Reporting of results	There is no selective reporting of results.	Y	Y	P	Y	Y	U	Y	U	U	P	Y	Y
Statistical analysis and presentation summary	The statistical analysis is appropriate for the design of the study, limiting potential for presentation of invalid or spurious results.	*L*	*L*	*L*	*L*	*L*	*M*	*L*	*M*	*M*	*M*	*L*	*L*

Abbreviations: H, high; L, low; M, moderate; N, no; N/A, not applicable; P, partial; U, unsure; Y, yes.

### 3.3 Socio‐Economic Status

Five studies reported socioeconomic status [22, 28, 29, 31, 33]. Four of these studies measured socioeconomic status with Index of Relative Socio‐Economic Disadvantage (IRSD) in which three studies used the participants' postcodes to calculate their IRSD [29, 31, 33]. One study used IRSD for each local government area (LGA) across Victoria and ranked them from most to least disadvantaged [22]. One study used Socio‐Economic Indexes for Areas (SEIFA) data from Queensland Government Statistician's Office and LGAs to compare the socioeconomic disadvantage between three LGAs in Queensland [28].

For DFU healing outcomes, Tehan et al. [33] found IRSD to be statistically significant for DFU healing with an odds ratio of 1.01 (95% CI 1.01–1.02) where the less disadvantaged participants had higher likelihood of healing. The mean IRSD of the healed group was 978.14 compared to the nonhealing group mean of 955.76 (*p* = 0.01). Jia et al. [31] found no association between IRSD and occurrence of DFU infection during healing, whereas Bergin et al. [22] reported rates of cellulitis and osteomyelitis in those with DFU to be lower in people living in less socioeconomically disadvantaged regions (Table 4).

For amputation outcomes, one study reported major amputation (below‐ and above‐knee) was more likely for people living in more socio‐economically disadvantaged regions [28]. Four‐year adjusted mean amputation rates were double for the most disadvantaged LGA (Logan 14/100,000) compared to the least disadvantaged LGA (Brisbane 7/100,000) [28]. Similarly, Bergin et al. [22] reported a higher rate of major amputation (below‐ and above‐knee) for more disadvantaged areas in Victoria (Table 4). However, Tehan et al. [29] did not find IRSD to have a statistically significant relationship with rates of minor or major amputation in people with DFU in Newcastle, New South Wales (Table 4).

### 3.4 Remoteness

Five studies reported on the effect of remoteness on DFU healing and amputation [23, 27, 30, 31, 32]. Three of the studies utilised Accessibility/Remoteness Index of Australia to define remoteness of the participants [23, 31, 32], one study used the Modified Monash Model (MMM) [30] and one classified remoteness by participants' usual place of residence without further definition [27].

For DFU outcomes, Zhang et al. [32] investigated time to healing in 65 outpatient Diabetes Foot Service sites across 15 Hospital and Health Service regions in the Queensland, Australia. They found living in regional or remote areas was independently associated with longer time‐to‐ulcer‐free (*p* < 0.05) (Table 4). However, Jia et al. [31] did not find any statistically significant association between rates of DFU infection and remoteness (Table 4).

For amputation outcomes, study findings were mixed. Two studies, found rates of lower extremity amputation, were not statistically significant between those living in remote areas compared to those living in regional or metropolitan areas [23, 30] (Table 4). However, O'Rourke et al. [27] found diabetes‐related major amputation was significantly associated with remoteness but only in those participants who identified as First Nations (Table 4).

### 3.5 Health Care Service Access

Seven studies reported on access to healthcare services [24, 25, 26, 27, 28, 29, 32]. Of these studies, DFU outcomes reported include rates of amputation (*n* = 5) [25, 26, 27, 28, 29], occasions of service for DFU complications (*n* = 3) [24, 25, 26], DFU healing times (*n* = 2) [25, 32], limb salvage (*n* = 1) [25] and survival rates (*n* = 1) [25] (Table 4).

Macfarlane et al. [25] found a statistically significant decrease in time to ulcer healing (*p* = 0.021), increase in incidence of vascular intervention (*p* = 0.002) and decrease in patient mortality (*p* = 0.045) after an interdisciplinary high risk foot service (HRFS) was implemented in 2018 (Table 4). In contrast, in two New South Wales tertiary hospitals in Sydney, Manewell et al. [26] did not find statistically significant differences for rates of DFU‐related hospital admission for diabetes‐related minor or major amputation between people known to the HRFS and new patients. Similarly, in Far North Queensland O'Rourke et al. [27] did not find statistically significant differences in diabetes‐related amputation between individuals attending HRFS and those who were not.

In relation to other aspects of healthcare access, Soonarane et al. [28] found that in the Logan LGA in Queensland, the majority of amputations were performed outside Logan LGA suggesting inadequate local service resources. Similarly, Tehan et al. [29] found increased odds of amputation with every 50 km distance from a HRFS [odds ratio 1.14 (95% CI 1.02–1.24) and *p* = 0.02] in Newcastle, New South Wales. For Queensland Diabetic Foot Services sites, Zhang et al. [32] found median time to ulcer‐free days to be significantly shorter with recent DFU treatment by a podiatrist compared to nil treatment by podiatrist (111 days compared to 193 days *p* < 0.05) (Table 4).

## 4 Discussion

This systematic review identified 12 studies investigating the relationship of SDH and outcomes for diabetes‐related foot disease (DFD), including foot ulcer healing and amputation, in Australia. This included five studies investigating socioeconomic status [22, 28, 29, 31, 33], five studies reporting on the effect of geographical remoteness [23, 27, 30, 31, 32] and seven studies investigating the effect of access to healthcare services [24, 25, 26, 27, 28, 29, 32].

Studies included in this systematic review tended to report that increasing socioeconomic disadvantage may contribute to lower likelihood of DFU healing and greater likelihood of amputation. Two studies included in this systematic review investigating DFU found a higher IRSD score was associated with greater likelihood of healing [33] and lower risk of DFU infection [22]. Another two studies investigated amputation outcomes and reported greater likelihood of major amputation was associated with greater socioeconomic disadvantage [22, 28]. This is consistent with findings from national data sets that have demonstrated increased risk of type 2 diabetes and diabetes‐related end stage renal disease for those in lower SEIFA deciles (indicating greater disadvantage) [34, 35]. For example, a person in the lowest SEIFA decile is more than twice as likely to have diabetes than a person in the highest SEIFA decile [34]. However, our review has also shown that the data examining the relationship between socioeconomic status and DFU amputation outcomes is limited and restricted to three geographical areas in Australia, including one area in regional Victoria, one area in regional NSW and one across three local government areas in Queensland [22, 28, 29]. Of the studies measuring socioeconomic status, one study used SEIFA [28] and the other four studies used the IRSD [22, 29, 31, 33]. As a measure of relative disadvantage only, the IRSD does not extrapolate to relative socioeconomic advantage, and as an area‐based measure has been reported to be problematic as a measure of individual or sub‐group disadvantage due to the potential for disparate communities to be included in the one area [36]. Furthermore, two studies conducted in the Hunter New England region of NSW reported that the mean IRSD scores for both groups, those experiencing the outcome (i.e., DFU healing or amputation) and those who did not, were relatively disadvantaged (IRSD < 1000) which is consistent with relative socioeconomic disadvantage across that region [29, 33]. This may explain the small positive relationship between increased likelihood of healing and a higher IRSD score (OR 1.01 and *p* < 0.01) and the absence of a statistically significant relationship between IRSD and amputation outcomes reported in these studies [29, 33].

Tehan et al. (2022) found increasing distance from a HRFS in regional New South Wales was incrementally associated with higher likelihood of amputation [29]. In Australia, it is well established that people living in rural and remote areas have worse general health outcomes compared to those in metropolitan areas, particularly in relation to chronic disease burden [37]. Generally, healthcare service delivery is impeded by limited workforce capacity in rural and remote areas, a lack of resources and difficulty in recruiting health practitioners to these areas [38, 39]. Location of the service and the individual is of particular relevance in Australia due to large geographical areas and low population density which contributes to irregular or absent health service provision in some rural and remote areas. Inadequate healthcare service access may be compounded by limited transport availability, particularly in areas of greater socioeconomic disadvantage [40, 41]. Although these are common barriers to accessing healthcare, they are specifically relevant to DFD where urgent care is frequently required and delayed treatment increases the risk of severe outcomes [42, 43].

Reflecting these challenges, the 2021 Australian DFD guidelines provide specific implementation considerations to support care provision for people living in rural and remote areas [44]. Nevertheless, our review shows that based on the limited data, the extent of the relationship between geographical remoteness and DFU healing and amputation outcomes remains difficult to determine. Current data are restricted to Queensland and the Northern Territory [23, 27, 30, 31, 32]. Across Queensland, DFU outcomes, including time to being ulcer‐free and ulcer recurrence, were found to be poorer for those living in a regional or remote areas [27, 30, 31, 32]. Similarly in other systematic reviews, amputation outcomes were worse for those living in remote areas in Far North Queensland and the Northern Territory [45, 46, 47]. Of note, this relationship was not upheld for those undergoing further amputation following a minor amputation in a recent study from Townsville, Queensland with the authors suggesting improved rural podiatry access and endocrinology telehealth services available in the state potentially contributed to their findings [30]. It is also unclear if the preceding minor amputation facilitated ongoing access to podiatry care as these outcomes related to risk of subsequent or additional amputation as opposed to initial presentation.

Current international guidelines for the prevention and management of DFD recommend DFU management by a multidisciplinary foot care team [18, 42]. In Australia, the National Association of Diabetes Centres has introduced national accreditation standards for HRFS to support access to high quality multidisciplinary diabetes foot care with development of such services ongoing across the country [48]. Consistent with guideline recommendations, in this present systematic review, there was evidence of implementation of a HRFS decreasing DFU time to healing, increasing likelihood of revascularisation and reducing mortality [25]. Interestingly, across several studies in metropolitan and regional areas, no difference in amputation rates between those known to HRFS and new presentations was reported [26, 27]. However, for Manewell et al. [26], only 18% of individuals undergoing amputation were known to the HRFS and only 9% had previously attended podiatry services. This was consistent with low rates of HRFS attendance prior to minor amputation reported by Linton et al. [24] and highlights the need for referral pathways to be well established with community care providers and known to relevant health professionals as well as healthcare consumers [49].

## 5 Future Directions

Surprisingly, this systematic review found relatively few studies investigating the relationship between SDH and DFU and amputation outcomes. Data were restricted to a small number of geographical regions and demonstrated that relevant SDH for DFU outcomes are variable according to the location and population involved. International research has demonstrated factors, such as increasing age, financial insecurity, lack of social support and clinical bias, towards people of low socioeconomic status may impact outcomes of DFD [50, 51, 52]. Further investigation is required across the scope of SDH, particularly in regions of Australia where there are high rates of DFU and amputation, to better inform comprehensive service delivery for both prevention and management of DFD.

## 6 Limitations

This systematic review is strengthened by a robust search strategy and the inclusion of several electronic databases. However, restricting the eligibility to published original studies may have resulted in reports from health services or other grey literature not being captured. We followed the PRISMA guidelines for systematic reviews and prospectively registered this systematic review with Prospero. The lack of meta‐analysis and limited geographical coverage of the included studies also limits the extent to which the study findings can be collectively interpreted and generalised. The authors recognise that social inclusion and non‐discrimination are important SDH and despite the lack of data retrieved in this review acknowledge the substantial and ongoing negative impacts of discrimination on health outcomes for minority groups in Australia including for First Nations Peoples.

## 7 Conclusions

This systematic review suggests that an increase in socioeconomically disadvantage adversely affects the likelihood of DFU healing. Although limited in geographical coverage, an increase in socioeconomic disadvantage appears to be associated with greater likelihood of amputation. Implementation of HRFS may see the effect of decreasing DFU healing time and mortality and increase the likelihood of revascularisation where required. However, there is a lack of consistency in reporting for HRFS utilisation and DFU and amputation outcomes. Inconsistency in the current available data and limited geographical coverage also inhibit the establishment of relationships between geographical remoteness and DFU healing and amputation. Further research on the contribution of SDH on DFU healing outcomes using a more consistent approach and standardised measurement is required. Further investigation is also warranted to determine the effects of other social and cultural determinants of health on DFU healing.

## Author Contributions

**Tsz Long Chan:** conceptualization, formal analysis, investigation, methodology, validation, writing – original draft, writing – review and editing. **Sean Sadler:** conceptualization, data curation, formal analysis, funding acquisition, investigation, methodology, project administration, resources, supervision, validation, visualization, writing – original draft, writing – review and editing. **Angela Searle:** conceptualization, investigation, methodology, supervision, writing – original draft, writing – review and editing. **Kym Hennessy:** investigation, methodology, supervision, validation, writing – original draft, writing – review and editing. **Sean Lanting:** investigation, methodology, supervision, validation, writing – original draft, writing – review and editing. **Vivienne Chuter:** conceptualization, data curation, formal analysis, investigation, methodology, project administration, resources, supervision, validation, visualization, writing – original draft, writing – review and editing.

## Funding

The authors have nothing to report.

## Ethics Statement

The authors have nothing to report.

## Consent

The authors have nothing to report.

## Conflicts of Interest

The authors declare no conflicts of interest.

## Data Availability

All data generated and analysed for this study are available in this systematic review or the original studies included.
